# HER2 overexpression and amplification is present in a subset of ovarian mucinous carcinomas and can be targeted with trastuzumab therapy

**DOI:** 10.1186/1471-2407-9-433

**Published:** 2009-12-10

**Authors:** Jessica N McAlpine, Kimberly C Wiegand, Russell Vang, Bridgett M Ronnett, Anna Adamiak, Martin Köbel, Steve E Kalloger, Kenneth D Swenerton, David G Huntsman, C Blake Gilks, Dianne M Miller

**Affiliations:** 1 Department of Gynaecology and Obstetrics, University of British Columbia, Vancouver, BC, Canada; 2 Center for Translational and Applied Genomics, BC Cancer Agency, British Columbia, Canada; 3 Department of Pathology, Johns Hopkins University School of Medicine, Baltimore, MD, USA; 4 Department of Pathology and Laboratory Medicine, University of British Columbia, Vancouver, British Columbia, Canada; 5 Department of Medical Oncology, BC Cancer Agency, Vancouver, BC, Canada

## Abstract

**Background:**

The response rate of ovarian mucinous carcinomas to paclitaxel/carboplatin is low, prompting interest in targeted molecular therapies. We investigated HER2 expression and amplification, and the potential for trastuzumab therapy in this histologic subtype of ovarian cancer.

**Methods:**

HER2 status was tested in 33 mucinous carcinomas and 16 mucinous borderline ovarian tumors (BOT)). Five cases with documented recurrence and with tissue from the recurrence available for testing were analyzed to determine whether HER2 amplification status changed over time. Three prospectively identified recurrent mucinous ovarian carcinomas were assessed for HER2 amplification and patients received trastuzumab therapy with conventional chemotherapy.

**Results:**

Amplification of HER2 was observed in 6/33 (18.2%) mucinous carcinomas and 3/16 (18.8%) BOT. HER2 amplification in primary mucinous carcinomas was not associated with an increased likelihood of recurrence. The prospectively identified recurrent mucinous carcinomas showed overexpression and amplification of HER2; one patient's tumor responded dramatically to trastuzumab in combination with conventional chemotherapy, while another patient experienced an isolated central nervous system recurrence after trastuzumab therapy.

**Conclusion:**

HER2 amplification is relatively common in ovarian mucinous carcinomas (6/33, 18.2%), although not of prognostic significance. Trastuzumab therapy is a treatment option for patients with mucinous carcinoma when the tumor has HER2 amplification and overexpression.

## Background

The majority of ovarian mucinous tumors are borderline tumors or stage I carcinomas, and the prognosis, overall, for patients with early stage mucinous carcinoma is excellent. The prognosis in patients with spread beyond the ovaries, however, is extremely poor. Chemotherapy with paclitaxel and carboplatin is recommended for patients with metastatic mucinous carcinoma, but response rates are considerably lower than are observed in other subtypes of epithelial ovarian cancer (EOC) [1-6]. At present no superior alternative treatment options exist.

HER2 is a member of the epidermal growth factor family of tyrosine kinase receptors. Activation of HER2 triggers a cascade of cellular responses, impacting cellular proliferation, angiogenesis and metastasis [7-9]. Amplification and overexpression of HER2 is seen in approximately 15% of breast carcinomas and is associated with a poor prognosis [10-14]. Adjuvant therapy using a monoclonal antibody against HER2 protein (trastuzumab) is effective alone and in combination with conventional cytotoxic chemotherapy in patients whose breast carcinomas have amplification of HER2 [15-18]. In contrast, the significance of HER2 overexpression and amplification in EOC is less well understood. The reported frequency of HER2 overexpression in EOC ranges from 5-66% [19-23], although more recent studies using validated techniques for detection of HER2 overexpression or amplification have consistently shown results at the low end of this range [21,22]. Clinical response to single agent trastuzumab in EOC has been disappointing. In a series of 41 patients with HER2 overexpressing EOC, identified from a series of 837 EOC tested for HER2 expression, there was only one complete responder and two partial responders for an overall response rate of 7.3% and a median progression-free interval of two months [19]. In this series, HER2 expression was determined by immunohistochemistry (IHC) only, and none of the patients in this series had carcinomas of mucinous subtype.

There has been an increasing appreciation of the molecular differences between the different histologic subtypes of EOC [24-26]. Differences in initial presentation, metastasis, response to therapy, and overall prognosis have been described and there has been criticism of the conventional approach of treating EOC as one entity [27]. Most series analyzing HER2 expression in EOC have not performed subtype analysis based on histology and often have poor or absent representation of mucinous carcinoma [19,20,22,23,28].

Given the absence of data on mucinous ovarian tumors and HER2 expression, inference may be permitted based on histological and immunohistochemical similarities between mucinous ovarian tumors and tumors of the upper gastrointestinal tract [29-31]. Activity of trastuzumab has been demonstrated in preclinical models of gastric and esophageal cancers [32-35]; approximately 7-15% of gastroesophageal adenocarcinomas show amplification of HER2. This prompted our investigation of HER2 expression in patients with recurrent mucinous EOC. Our objectives in this study were 1) to look for HER2 protein overexpression (IHC) and gene amplification (FISH) in our current and a historical patient population of patients with mucinous EOC and mucinous borderline ovarian tumors (BOT), 2) examine the correlation between HER2 immunostaining and amplification, 3) determine if HER2 expression or amplification status changed from the time of initial presentation to recurrence, 4) treat patients with recurrent mucinous ovarian carcinoma with trastuzumab, when the tumor has HER2 amplification and overexpression, and monitor for response to treatment.

## Methods

### Case Selection

Following Institutional Review Board approval the following cases were identified: 1) a cohort of 34 cases of mucinous carcinoma from 1984-2000 in British Columbia (BC); these were identified as part of a population-based review of cases of ovarian carcinoma who had no microscopic residual disease after primary surgery. This cohort has been described previously [36] and the 34 mucinous carcinomas are part of a tissue microarray consisting of 541 cases of ovarian cancer, 2) three mucinous carcinomas and seven mucinous BOT collected as part of our Ovarian Tumor Bank in BC, since 2000, 3) three archival cases from a previously published series on mucinous ovarian carcinomas from our institution [37], and 4) 15 mucinous BOT cases, gastrointestinal type, from the pathology archives of Johns Hopkins University School of Medicine collected from their institution (n = 12) or as consults from other institutions (n = 3) during the period between 1994-2005. Three patients with recurrent mucinous carcinoma and HER2 amplification were treated with a combination of HER2 targeted therapy (trastuzumab) and platin-based chemotherapy and followed prospectively. Response was based on serial examinations, tumor markers, and CT imaging (RECIST criteria) and written informed consent was obtained for publication of associated text and images. A copy of the written consent is available for review by the Editor-in-Chief of this journal.

### Immunohistochemistry

IHC was performed on either whole sections, or for the retrospective, population based series, on tissue microarray slides in which duplicate 0.6 mm cores from each case were present in the array. Four micron thick sections were immunostained on a Ventana Benchmark XT staining system (Ventana Medical Systems, Tucson, AZ, USA). Sections were deparaffinized in xylene, dehydrated through three alcohol changes and transferred to Ventana Wash solution. Heat antigen retrieval was used. Endogenous peroxidase activity was blocked in 3% hydrogen peroxide. Slides were then incubated with rabbit monoclonal anti-HER2 Ab (clone SP3) at a dilution of 1:50, at 37°C for 32 min, and developed with a proprietary Ventana amplification reagent kit followed by DAB chromogen. Finally, sections were counterstained with hematoxylin and mounted. HER2 was scored visually according to the ASCO/CAP guidelines [38]: 0 or 1+ (negative): no staining or incomplete membrane staining in > 30% of tumor cells; 2+ (weakly positive, equivocal): strong, complete membranous staining in < 30% cells or weak to moderate heterogeneous staining in > 10% cells); 3+ (strongly positive: strong complete membrane staining in > 30% of tumor cells). All cases were reviewed and scored by one pathologist (CBG). Tissue cores that were missing, or were otherwise uninterpretable were not included in the analysis.

### Fluorescence in situ hybridization

Six-micron sections of the TMA slides were hybridized with probes to LSI^® ^Her-2/neu and CEP^® ^17 with the PathVysion™ HER-2 DNA Probe Kit using a modified protocol. Briefly, slides were baked overnight at 60°C, deparaffinized and dehydrated. Pre-treatment washes included 10 mM citric acid buffer (pH 6.0) (80°C, 45 minutes), 2× SSC twice (5 minutes each), distilled water (1 minute). Slides were protease treated at 37°C for 12 minutes, washed with 2×SSC twice (5 minutes each), dehydrated and air-dried, then counterstained with DAPI and visualized on a Zeiss Axioplan epifluorescent microscope. Analysis of FISH signals was performed using MetasystemsTM automated image acquisition and analysis system, Metafer (Metasystems, Altlussheim, Germany). This FDA-approved, automated system scores FISH signals by employing specific measurement algorithms to detect and quantify clustered signals. A high correlation between manual and automated scoring of FISH signals has been previously reported [39]. Average copy number for each probe was calculated and the amplification ratio (ratio between the average copy per cell for HER2 and the average copy for centromere 17) determined. Amplification ratios > 2.2 are considered positive [38]. Tumors that failed to hybridize were not included in the analysis.

### Statistical analysis

Tests for heterogeneity were performed for the parameters: age, stage, grade, residual disease, exposure to previous chemotherapy, and the mean follow-up time with regard to both progression and overall survival for the HER2 positive and HER2 negative cohorts utilizing the Welch ANOVA, or the Pearson χ^2 ^statistic as appropriate. Progression free survival (PFS) is defined as the time from surgery to the first clinical evidence of recurrence ("chemical" recurrences i.e., tumor marker elevations not included). Overall survival (OS) time is defined as the time from surgery until death from any cause, or until the last date of follow-up. Kaplan-Meier survival analyses and the log-rank test were used to assess the impact of various clinicopathologic parameters and HER2 amplification on PFS and OS time.

## Results

**Figure 1:**
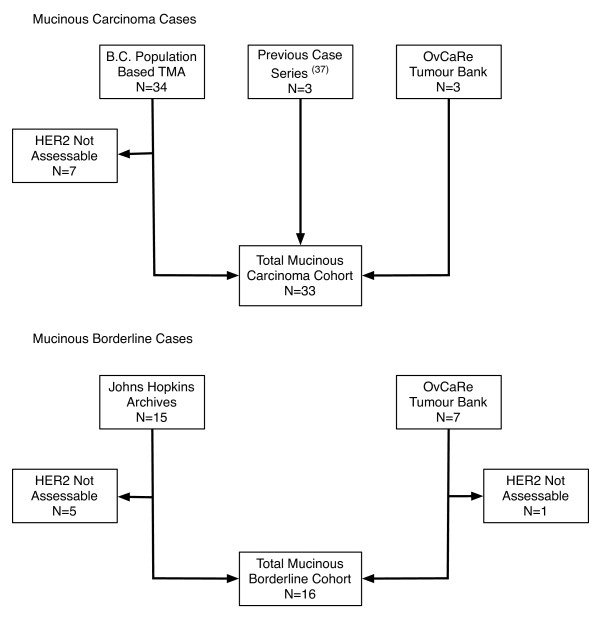
**Flowchart outlining the process of case identification for our retrospective series of mucinous ovarian cancers and mucinous borderline ovarian tumors**.

Immunostaining and FISH data were available for 33 cases of mucinous carcinoma and 16 cases of mucinous BOT (Figure 1). Loss of cases from the original pool of 40 carcinomas was primarily due to use of tissue microarrays where small sample size with few tumor cells or loss of tissue after digestion result in inability to assess amplification [39]. Demographic and clinicopathologic data for the mucinous carcinomas with and without HER2 amplification are shown in Table 1. Tumor grade was the only parameter shown to be associated with progression free (PFS) (p < 0.001) and overall survival (OS) time (p < 0.001). HER2 overexpression by IHC was seen in five of the carcinomas (3+ in four cases, 2+ in one case). There was high-level HER2 amplification (HER2/CEP17 ratios > 5) in six cancer cases (6/33 = 18.2%), including the five cases with HER2 overexpression. However, one case had discordant IHC and FISH results (IHC score of 0, FISH HER2/CEP ratio of 6.7) (Table 2). Of sixteen mucinous borderline tumors, three (3/16 = 18.8%) demonstrated HER2 amplification (HER2/CEP17 ratios of 3.1-3.3). One case of BOT showed discordant results with an IHC score of 0 and HER2/CEP ratio of 2.4 (Table 3).

**Table 1:** Demographics and clinicopathologic parameters for the 33 mucinous ovarian carcinoma cases identified retrospectively.

Parameter		HER2+	HER2-	p-value
Age (years)		48.0 (31-72)	51.4 (18-76)	0.62_a_

Stage	I	67% (N = 4)	67% (N = 18)	0.88_b_

	II	33% (N = 2)	29% (N = 8)	

	III	0% (N = 0)	4% (N = 1)	

Grade	1	50% (N = 3)	30% (N = 8)	0.55_b_

	2	50% (N = 3)	63% (N = 17)	

	3	0% (N = 0)	7% (N = 2)	

Residual Disease	No	100% (N = 6)	100% (N = 27)	NR

Prior Chemotherapy	No	100% (N = 6)	100% (N = 27)	NR

Mean Progression Free Survival (years)		7.90 (3.20 -- 11.35)	5.15 (0.17 -- 20.4)	0.12_a_

Mean Overall Survival (years)		7.90 (3.20 -- 11.35)	5.43 (0.35 -- 20.4)	0.16_a_

Progression free survival (PFS) is defined as the time from surgery to the first clinical evidence of recurrence. Overall survival (OS) is defined as the time from surgery until death from any cause, or until the date of last follow-up. All times are given in years

a Comparisons across HER2 status computed with the Welch ANOVA test

b Comparisons across HER2 status computed with the Pearson Chi Square Statistic

NR Not reported due to equivalence

**Table 2:** Immunohistochemistry (IHC) and fluorescence in-situ hybridization (FISH) results for HER2 protein expression and gene amplification respectively with amplification (in bold) observed in 6/33 (18.2%) mucinous carcinomas.

ID	IHC (HER2)	HER2/CEP 17 Ratio	Patient Outcome	IHC/FISH concordance
V1	0	0.8		

**V2**	**3**	**8.0**		

V3	0	1.0		

V4	0	1.1		

**V5**	**3**	**5+**		

**V6**	**3**	**5.5**		

V7	0	1.1		

V8	1	1.9		

V9	0	1.2		

**V10**	**0**	**6.7**		**Discordant**

V11	0	1.0		

V12	0	0.7		

V13	0	1.0		

V14	0	0.9	Recurrent	

V15	0	1.1		

V16	0	1.1		

V17	0	1.4	Recurrent	

V18	0	1.3	Recurrent	

V19	1	0.8	Recurrent	

**V20**	**2**	**6.2**		

V21	0	1.5	Recurrent	

V22	0	1.2		

V23	0	1.2		

V24	0	1.7	Recurrent	

V25	0	1.1		

V26	0	0.9		

V27	0	1.0	Recurrent	

**V28**	**3**	**5.1**		

V29	1	1.3		

V30	0	1.0		

V31	0	1.2	Recurrent	

V32	0	1.4	Recurrent	

V33	0	1.0	Recurrent	

**Table 3:** Immunohistochemistry (IHC) and fluorescence in-situ hybridization (FISH) results for HER2 protein expression and gene amplification respectively with amplification (in bold) observed in 3/16 (18.8%) mucinous borderline tumors of the ovary.

ID	IHC (HER2)	HER2/CEP 17 Ratio	IHC/FISH concordance
VB1	0	0.9	

VB2	0	0.8	

VB3	0	0.8	

VB4	1	1.2	

VB5	0	1.2	

VB6	0	1.0	

**J1**	**3**	**3.2**	

J2	0	0.8	

J3	0	1.2	

J4	0	1.1	

J5	0	1.1	

J6	0	1.2	

**J7**	**0**	**2.4**	**Discordant**

**J8**	**2**	**3.1**	

J9	0	1.3	

J10	0	1.3	

**Figure 2:**
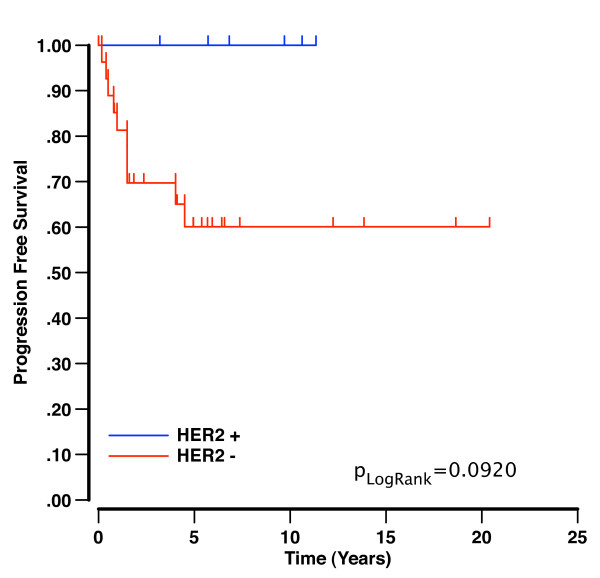
**Kaplan-Meier survival curves demonstrating that the presence of HER2 amplification in primary mucinous carcinomas is not of prognostic significance with respect to disease recurrence**.

We then looked at any recurrences with tissue available for FISH and IHC, to determine if HER2 expression levels/copy number changed in the recurrence. Seven patients whose tumor was represented on the TMA and all three of the cases from the previously published case series [33] developed recurrent disease. None of these 10 cases that recurred had initial HER2 amplification. Tissue specimens were available for testing in 2/10 recurrences, neither of which demonstrated HER2 immunoreactivity or amplification (HER2 amplification ratio of 1.2 and 0.77, respectively). Among the cases represented on the TMA, there were no recurrences in the six patients with HER2 amplification, and seven recurrences in the 27 cases where HER2 was not amplified. There was no significant difference in prognosis associated with HER2 amplification at the time of diagnosis (p_Log-Rank _p = 0.0920; (Figure 2).

Of the three cases of recurrent mucinous carcinoma identified prospectively, all showed strong HER2 expression and amplification at the time of recurrence and were treated with a combination of conventional chemotherapy and trastuzumab. These cases are described in detail below.

### Case 1

**Figure 3:**
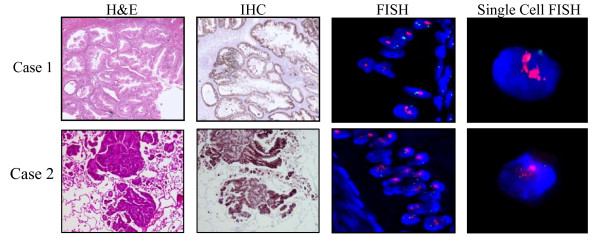
**HER2 immunostaining and FISH of tumors from cases 1 and 2 (Case 2-sample from lung), who subsequently received trastuzumab either alone or in combination with conventional chemotherapy**. Each tumor shows strong immunoreactivity for HER2 and amplification by FISH (HER2 probe -- red, CEP17 probe -- green).

**Figure 4:**
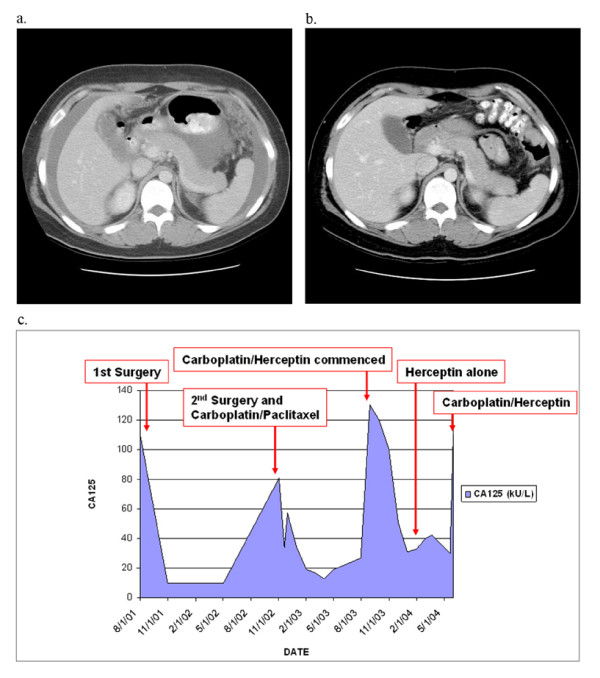
**Computed tomography images of Case 1**. The first image (a.) was taken four months after completion of (surgery and) chemotherapy treatment for her first recurrence. Imaging had been ordered for increased gastrointestinal symptoms and an elevation in her tumor markers. Ascites and omental disease are noted. Carboplatin and trastuzumab were commenced with a dramatic response (b. resolution of ascites and omental nodules) seen after only three cycles. Graphic representation of CA125 levels (c.) also demonstrates a drop in CA125 levels after the initiation of carboplatin and trastuzamab therapy and stable CA125 levels during trastuzamab monotherapy for at least three cycles.

The first patient with recurrent mucinous carcinoma initially presented at age 19 with irregular periods, pelvic pain, increased abdominal girth, and an elevated CA125 of 110 kU/L (other markers normal). Imaging revealed a 15 cm mass and ascites. She underwent surgical staging including unilateral salpingoophorectomy (USO), appendectomy, omentectomy, peritoneal biopsies, and washings. Pathology reported a 20 × 15 × 14 cm mucinous BOT, intestinal type with focal inatraepithelial carcinoma of the ovary, all other specimens negative, stage Ia. She was observed and did well until 15 months later when she was noted to have an elevation in her CA125 to 81 kU/L. A CT scan revealed ascites and a mass in the contralateral ovary. She underwent USO and multiple biopsies. Pathology showed a mucinous borderline tumor of the ovary with intraepithelial carcinoma, but there were now implants of invasive mucinous carcinoma on the peritoneal surfaces. She received carboplatin and paclitaxel (CP) for six cycles, with normal CA125 throughout but again recurred four months after completion of therapy, based on reaccumulation of ascites, omental disease, elevated CA125 (130 kU/L), and abdominal symptoms. Pathology review of her first recurrence was performed to assess for molecular markers. This revealed the overexpression and amplification of HER2 (IHC 3+, HER2/CEP ratio 7.2) (Figure 3) and trastuzumab (6 mg/kg) was given in addition to single agent monthly carboplatin (600 mg/m2). A dramatic response, based on imaging and tumor markers, was noted after three cycles (Figure 4) and she completed a total of six cycles of this combination. She then received trastuzumab alone for three cycles with stable disease after which her markers began to rise and ascites and omental disease were seen on CT scan. Carboplatin was reintroduced but her markers continued to increase and she was changed to gemcitabine in combination with trastuzumab. Her CA125 level dropped from 1800 to 180 kU/L after the first cycle but she developed signs and symptoms of large bowel obstruction. She was taken to surgery for necrotic tumor in her cecum and splenic flexure and underwent a hemicolectomy and debulking without complications. She continued on gemcitabine and trastuzamab for six cycles with stable markers (CA125 range 50-210 kU/L). She progressed and failed three other traditional chemotherapy agents (capecitabine, liposomal doxorubicin, and etoposide) before ultimately succumbing to her disease. She died 50 months from time of diagnosis secondary to respiratory distress with massive intractable pleural effusions and pulmonary emboli.

### Case 2

The second patient was a 33yo taken to the operating room for a 10 cm mass suspected to be benign. There was intra operative rupture of thick mucus within the abdominal cavity. RSO and washings were performed. Final pathology revealed a FIGO grade 2 invasive mucinous carcinoma with destructive stromal invasion, and normal fallopian tube. She was fully staged (USO, appendectomy, biopsies, washings) at a second procedure one month later, with all specimens negative, Stage Ic. She received three cycles of CP followed by pelvic and whole abdominal radiation. She recurred 40 months later with a large pulmonary metastasis and subcarinal lymphadenopathy. She was initially deemed unresectable and received CP for four cycles and achieved a partial remission. The CA 19-9 had also decreased from a high of 1000 kU/L to 80 kU/L pre-thoracotomy. She underwent right middle and lower lobectomy. Immunohistochemistry of her tumor at this time revealed 3+ positivity for HER2 protein and a HER2/CEP17 ratio of 7.5 (Figure 3). She was then changed to trastuzumab monotherapy, which she took for a total of 5 cycles (6 mg/kg for three weeks) and remained without clinical evidence of disease and with normal tumor markers. One month after the discontinuation of trastuzumab therapy she began experiencing severe headaches, neck spasms and vomiting. A CT scan of the head revealed multiple bilateral brain metastases (prior CT's of the head negative, within six months), predominantly in her frontal lobes with possible interventricular extension. She was given whole brain radiation, 2000 cGy prescribed to the midplanes in five fractions. Despite radiation the patient developed progressive intracranial tumor without evidence of disease elsewhere. She died less than three months after discovery of her brain metastases, 56 months from initial diagnosis.

**Figure 5:**
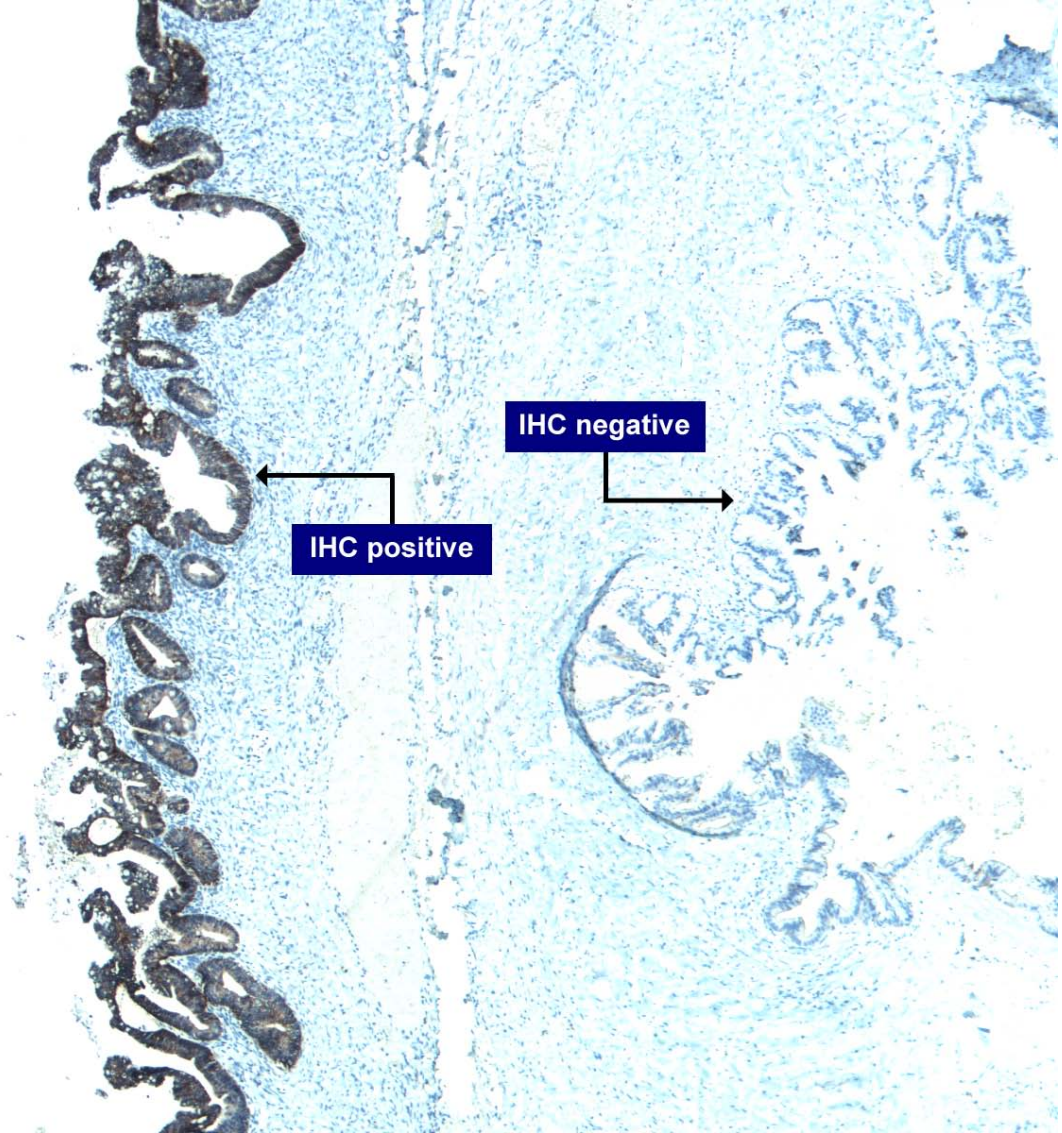
**Prospectively identified Case 3: Tumor from initial presentation classified as a mucinous borderline ovarian tumor (BOT) and shows a discrete area of HER2 positivity in what is predominantly a HER2 negative tumor**.

In the third case, evaluation of her response to trastuzumab alone and in combination with platin-based chemotherapy was not possible by RECIST criteria (not imaged pre/post therapy and inconsistent tumor marker assessment). Interestingly, evaluation of her primary presentation, first recurrence and second recurrence showed an apparent change in HER2 amplification status. Careful re-analysis of the primary tumor identified an area of tumor heterogeneity. The primary tumor was predominantly HER2 negative with only focal HER2 expression (Figure 5). The areas showing overexpression also showed HER2 amplification (data not shown). In the recurrent specimens (28 and 57 months from initial diagnosis) there was diffuse HER2 overexpression and amplification.

## Discussion

Development of treatments for rare tumors is challenging. The NCI State of the Science meeting on ovarian cancer in 2005 recognized the need for separate trials for ovarian mucinous carcinoma, a rare subtype of EOC that responds poorly to conventional chemotherapy [23,27]. An increased understanding of the importance of histologic subtype in EOC has resulted in an increased emphasis on understanding the molecular changes leading to the development of tumor subtypes with the goal of targeted therapy specific to each subtype. Success with this strategy is evident in breast cancer and there is increasing evidence from preclinical models of gastroesophageal cancers that HER2 can be targeted in this disease [28-30,32-34]. Mucinous EOC resembles adenocarcinoma of the gastroesophageal region and molecular targeted therapy may also be indicated in appropriately selected cases of mucinous EOC.

Previously reported series investigating the prognostic implications of HER2 overexpression or HER2 targeted therapy in EOC included few or no cases of mucinous histology [19-23,28]. Varying techniques have been used to determine HER2 overexpression, often with less specific IHC assays, no FISH correlation, and inconsistent scoring/classification systems. Our series suggest that immunohistochemistry, FISH, and a scoring system similar to that used for breast cancer can be used for mucinous EOC and that there is good correlation between IHC and FISH results (2/49 or 4% discrepant, all with negative IHC and positive FISH). The correct interpretation for these cases with discordant IHC and FISH is not clear. The frequency of HER2 overexpression/amplification is higher than previously reported (18%) and these cases are candidates for molecular therapy

A dramatic response was observed in a patient with recurrent mucinous EOC showing amplification of HER2, treated with trastuzumab in combination with traditional chemotherapy after conventional therapy had ceased to work. The second prospectively identified case with HER2 overexpression and amplification received trastuzumab treatment, however she developed isolated intracranial tumor metastasis, a rare site of metastatic tumor in EOC. HER2 overexpression may provide tumor cells with increased metastatic aggressiveness thereby increasing the spread to sites such as the lungs and the central nervous system [40]. In breast cancer patients, isolated central nervous system metastases have been observed in 9-10% of patients receiving trastuzumab-based therapy [41]. The development of central nervous system metastases in these patients may occur due to increased patient survival times (i.e., brain metastases may become symptomatic as a result of an extended life span), and the inability of trastuzumab to penetrate the blood-brain barrier [40]. We postulate that this limitation in trastuzumab therapy explains the isolated brain recurrence in this patient who had complete resolution of her disease process in all other locations.

The prognostic implications of HER2 amplification in mucinous EOC or BOT have not been studied previously. None of the cases with HER2 overexpression or amplification identified in the retrospective case series experienced a recurrence. Determination of HER2 status at the time of diagnosis is unlikely to be a clinically relevant prognostic indicator. We believe, however, that assessment of HER2 status can provide valuable information in patients with advanced stage or recurrent mucinous EOC. For those patients whose tumors demonstrate overexpression and amplification of HER2, targeted therapy with trastuzumab (+/- conventional chemotherapy) can be considered. As seen in other cancers, HER2 heterogeneity was demonstrated in one of our mucinous ovarian carcinomas and repeat analysis of tumors of interest may be warranted.

## Conclusion

Prior investigations suggest HER2 amplification does not seem to be a significant event in epithelial ovarian cancers when analyzed across all histologic subtypes. However, we have demonstrated that in ovarian mucinous carcinomas HER2 amplification is relatively common (6/33, 18.2%), although not necessarily of prognostic significance. Response to conventional therapy is limited in this rare histologic subtype of EOC and trastuzumab therapy provides a treatment option for patients with mucinous carcinoma when the tumor has HER2 amplification and overexpression.

## Competing interests

Martin Köbel was supported through a non directed educational grant from Eli Lilly Canada.

## Authors' contributions

JM, DH, DM, KW and BG participated in the design, and coordination of the manuscript with JM, KW, and BG principally involved in its draft. JM, DM and KS provided clinical care and pertinent clinical information on the involved patients. KW interpreted the FISH results. BG, DH, and MK reviewed the pathology, scored the IHC, and BG and DH confirmed discrepant FISH results. AA performed the IHC. BR and RV provided cases and clinical histories from their institution, and shared their expertise in mucinous ovarian tumors. All authors read and approved the final version of the manuscript.

## Pre-publication history

The pre-publication history for this paper can be accessed here:

http://www.biomedcentral.com/1471-2407/9/433/prepub
